# Divergent evolutionary trajectories of male and female investment in the Brassicaceae

**DOI:** 10.1093/aob/mcaf176

**Published:** 2025-08-04

**Authors:** Mohammadjavad Haghighatnia, Marek Svitok, Marcus A Koch, Roswitha Schmickl, Clément Lafon Placette

**Affiliations:** Department of Botany, Faculty of Science, Charles University, Prague CZ-128 01, Czech Republic; Institute of Botany, The Czech Academy of Sciences, Průhonice 252 43, Czech Republic; Department of Biology and General Ecology, Faculty of Ecology and Environmental Sciences, Technical University in Zvolen, Zvolen 960 01, Slovakia; Department of Forest Ecology, Faculty of Forestry and Wood Sciences, University of Life Sciences Prague, Praha 6, Suchdol 165 21, Czech Republic; Plant Science and Biodiversity Centre, Slovak Academy of Sciences, Bratislava 845 23, Slovakia; Department of Biodiversity and Plant Systematics, Centre for Organismal Studies, Heidelberg University, Heidelberg 69120, Germany; Department of Botany, Faculty of Science, Charles University, Prague CZ-128 01, Czech Republic; Institute of Botany, The Czech Academy of Sciences, Průhonice 252 43, Czech Republic; Department of Botany, Faculty of Science, Charles University, Prague CZ-128 01, Czech Republic

**Keywords:** Sexual allocation, evolutionary response, phylogenetic constraints, mating system, ploidy level, pollinator abundance, Brassicaceae

## Abstract

**Background and Aims:**

Plants have colonized diverse habitats, with their ecological success depending on key adaptive traits. Among these, reproductive characteristics such as pollen and ovule production are critical. The allocation to sexual reproduction is likely shaped by diverse selective pressures and constraints. This study investigates the evolution of pollen and ovule production and its drivers in the Brassicaceae family.

**Methods:**

We analysed 158 Brassicaceae species, recorded pollen and ovule numbers, and used phylogenetic analyses to characterize the evolution of these traits. We then cultivated a subset of 66 species in a common garden and tested the association of pollen and ovule production with environmental conditions of original habitats.

**Key Results and Conclusions:**

Ovule production is strongly constrained by phylogeny, whereas pollen production is more evolutionarily flexible. Expectedly, recurrent mating system shifts played a role in this observation, as selfing species consistently produced fewer pollen grains but did not show significant changes in ovule number, supporting selection for reduced male investment in response to increased mating efficiency. In contrast, environmental associations with both traits were limited yet significant, with pollen and ovule production evolving according to different environmental pressures. Polyploidy did not influence the evolution of these traits. These phylogeny-informed findings refine our understanding of the evolutionary drivers of sexual allocation, demonstrating that male and female investment evolve under distinct selective pressures in hermaphroditic species.

## INTRODUCTION

The success of an organism depends on how effectively it allocates limited resources for growth, survival and reproduction, as defined by resource allocation theory (Charnov, 1982; Gillet and Gregorius, 2020). Sexual reproduction, in particular, involves trade-offs between male and female investment, influencing species-specific reproductive strategies (Charnov, 1982; Delph, 1999). In plants, reproductive investment involves balancing pollen and ovule production costs, which require different amounts of energy. Ovules are thought to require greater maternal resources, so their production is more limited, whereas pollen is likely produced at a lower cost (Charnov, 1982). Consistently, simulations indicate that successful fertilization requires greater reproductive investment in ovules than pollen, reinforcing that pollen is less costly to produce (Gillet and Gregorius, 2020). In addition, calorimetry in *Amaryllis vittata* showed that the energetic investment in total pollen and ovules per flower was comparable (Smith and Evenson, 1978), despite a greater number of pollen grains than ovules, and thus a lower energetic cost per pollen grain. Consequently, selection may favour different investment strategies for the male and female function. Species exhibit a wide range of ovule numbers, from uniovular taxa to those producing thousands per flower (Burd *et al.*, 2009; Friedman and Barrett, 2011; Wetzstein *et al.*, 2013). This variation reflects an evolutionary trade-off between offspring quantity and quality: producing more ovules reduces per-seed resource allocation, thereby influencing offspring fitness and survival (Baker, 1972; Greenway and Harder, 2007). Although pollen production is energetically cheaper, excessive investment leads to diminishing returns due to inefficient pollen transfer (Harder and Johnson, 2025), as only a small fraction reaches stigmas (Minnaar *et al.*, 2019). Short-term plasticity studies suggest that pollen production, however cheap, is still limited by environmental resources. For instance, Wilson Rankin *et al.* (2020) found that *Trifolium willdenovii* reduced pollen output under drought stress. Therefore, in resource-rich environments, selection may favour plants that invest more in pollen production, increasing pollination success and fertilization rates. Conversely, in resource-limited environments, excessive pollen production may be disadvantageous, as its effect on mating success is limited due to low pollen transfer efficiency (Minnaar *et al.*, 2019) and the energy cost would not compensate for the toll on survival. In these conditions, natural selection would favour plants that reduce pollen production, as lower reproductive costs improve survival and long-term reproductive success (Obeso, 2002; Edward and Chapman, 2011). Nevertheless, these expectations are contradicted by a recent modelling study showing that the evolutionarily optimal pollen production was not affected by the amount of resources available for reproduction (Bochynek and Burd, 2024), calling for additional investigation. Ovule production may follow a clearer trend, with greater investment in response to higher resource availability to maximize reproductive potential. Consistently, in *Embothrium coccineum* (Proteaceae) ovule production was positively associated with rainfall (Chalcoff and Aizen, 2016). The evolutionary pressures shaping pollen and ovule production may thus not be identical, as ovules require direct maternal investment in offspring (Yadegari and Drews, 2004; Phillips and Evans, 2020), while pollen disperses to achieve fertilization (Harder and Johnson, 2008; Ollerton *et al.*, 2011). This fundamental difference in parental investment suggests that the two traits may respond to environmental constraints in different ways, but this remains to be tested.

These distinct trajectories are also reflected in the factors influencing male and female reproductive success. Bateman’s principle predicts that male reproductive success is mating-limited, while female success is resource-constrained (Bateman, 1948; Moore and Pannell, 2011; Collet *et al.*, 2014). In plants, this means that increased pollen transfer enhances mostly male reproductive success (Queller, 1984), whereas female reproductive success remains stable independently of pollen transfer, as even a small pollen load can fertilize all ovules (Elle and Meagher, 2000; Collet *et al.*, 2014). Since only ∼5 % of pollen reaches conspecific stigmas (Gong and Huang, 2014; Minnaar *et al.*, 2019), selection may favour increased pollen production to compensate for inefficient transfer. Consistently, empirical studies have shown a negative relationship between pollen production and transfer efficiency (Gong and Huang, 2014). Another study indicates that pollen production is more responsive to pollinator availability (Harder and Johnson, 2023), whereas ovule investment may be constrained by maternal resource allocation. Thus, in pollinator-scarce habitats plants may produce more pollen as a consequence of selection on the male function for increasing mating opportunities. Interestingly, these observations and expectations collide with a recent modelling study suggesting that, in large panmictic populations at least, pollen production does not evolve to increase to compensate for low transfer efficiency even under pollen limitation (Bochynek and Burd, 2024), calling for further investigation.

Female reproductive success is often maximized with a single visit, making ovule production less responsive to pollinator availability. This is, however, different in the case of pollen limitation, and some researchers proposed ovule overproduction as a bet-hedging strategy against pollen receipt variability (Friedman and Barrett, 2009, 2011): in pollinator-scarce environments, uncertain pollen transfer increases reproductive failure risk, and thus producing more ovules may ensure seed production, particularly in fluctuating pollinator populations, seasonal climates or high-elevation habitats. Empirical studies yield some evidence in this direction. Burd *et al.* (2009) found ovule number positively correlated with stochastic pollination, suggesting selection for increased ovule production in unpredictable environments. Similarly, Arroyo *et al.* (2019) found an increase in ovule number in central Chilean alpine plants, supporting the idea that plants in high-altitude environments, where pollinator visits are less predictable, may produce a high number of ovules as a bet-hedging strategy. These observations point towards pollen limitation as a cause of female reproductive investment strategies to prevent reduced seed output. As an alternative strategy, the transition to selfing may ensure reproductive assurance despite limited mating opportunities, such as in populations at species range edges (Griffin and Willi, 2014). Convergently across angiosperm species, the transition to selfing is accompanied by a reduction in pollen production (Cruden, 1977; Tsuchimatsu and Fujii, 2022), assumed to be a consequence of efficient pollen transfer and thus relaxed selection on pollen production. On the contrary, the transition to selfing does not seem to have an impact on ovule production (Harder and Johnson, 2023).

Altogether, the impact of resource availability and pollination uncertainty on the evolution of male and female investment remains poorly understood in plants beyond specific cases. While past studies suggest that pollen and ovule production respond to both abiotic and biotic selection pressures, the relative influence of these factors in shaping reproductive allocation evolution is unresolved. Moreover, the extent to which the evolution of pollen and ovule production responds differently to these factors remains to be explored. Finally, a phylogeny-informed approach to these questions could give a broader view on the evolution of these traits. To address these gaps, we focus on the Brassicaceae family, which shows variation in habitat preference, mating system and ploidy level, and has a well-studied phylogeny (Nikolov, 2019; Al-Shehbaz, 2025). In this study we investigate (1) how constrained the evolution of pollen and ovule production is, (2) whether pollen and ovule production show an evolutionary response to environmental resources, and (3) how pollination assurance (via pollinator availability and transition to selfing) influences the evolution of these traits. Overall, we investigate how evolutionary constraints shape trait diversity by examining the interactions between stabilizing selection, ecological pressures and phylogenetic history in influencing reproductive investment in plants.

## MATERIALS AND METHODS

### Species sampling

For the phylogenetic analyses, pollen and ovule numbers were retrieved for 158 species from the Brassicaceae, spanning 71 genera and 29 tribes. These data originated from two different sources: our measurements (see below) and the literature (Supplementary Data Dataset S1).

For the association between environment and reproductive traits, a total of 66 species were cultivated at the Heidelberg Botanic Garden. For each cultivated species, two populations were selected, with two or three individuals per population (Supplementary Data Dataset S2).

### Plant material and measurements

For the 66 cultivated species, all plants were grown in outdoor greenhouses in Heidelberg University facilities (Germany) under non-limiting water and nutrient conditions. Perennial species were cultivated from spring 2018 and harvested for flower buds between September 2019 and March 2021. Annuals were cultivated for 1 year maximum prior to the same harvesting period. Flower buds close to anthesis were collected from the top part of the peduncle (three buds per individual), transferred to a fixing solution of ethanol:acetic acid (9:1) and kept at 4 °C overnight. The fixed buds were then washed with 90 % ethanol and stored in 70 % ethanol at 4 °C. In addition, buds from the same individuals were collected in teabags and dried, providing supplementary material for further analyses. Among the cultivated plants, 43 species, encompassing 25 genera and 14 tribes, had detailed locality data that were used for trait–environment associations (for a map showing the localities, see Supplementary Data Fig. S1).

For ovule number measurements, flower buds stored in 70 % ethanol were transferred to a clearing solution (66.7 % chloral hydrate, 25 % double-distilled water, 8.3 % glycerol) to render tissues transparent. The pistils were carefully dissected from the remaining floral parts (sepals, petals, anthers) and mounted on microscope slides. The ovules in each ovary were counted using a microscope equipped with differential interference contrast. Three buds per individual were measured to account for within-plant variation in ovule number.

For pollen measurements, dried flower buds (previously stored in teabags) were individually placed into 2-mL microtubes containing 95 % sulfuric acid (analytical grade, Penta Chemicals) and incubated at 37 °C for 10 d, dissolving all tissues except for pollen. After incubation, the tubes were centrifuged at 1000 *g* using a Centrifuge 5424 R (Eppendorf) and the supernatant was discarded. The pellet was resuspended in 1.5 mL of 0.01 % (v/v) Triton™ X-100 (Serva Electrophoresis GmbH), and this washing step was performed three times to remove any residual acid. The final pellet was then resuspended in 100 µL of Triton™ X-100 solution. After vortexing, 1 µL of the suspension was transferred to a BRAND^®^ counting chamber (BLAUBRAND^®^ Neubauer improved, Sigma–Aldrich). Under the microscope, pollen grains were counted in five 0.1-µL squares. The total pollen count per 100 µL was then calculated by multiplying by 10 the average number of pollen grains per square (to give the average number of pollen in 1 µL) and then multiplying the result by 100 (to adjust from 1 to 100 µL). Three buds per individual were measured to account for technical variation in pollen number.

### Abiotic and biotic environmental data

The geographical positions of species occurrences were extracted from the original collection records of the seeds from which the plants had been grown from the database of the Heidelberg Botanic Garden and Herbarium (HEID). Subsequently, the species occurrence data were complemented with altitude and climate data extracted from the WorldClim database version 2.1 (Fick and Hijmans, 2017). In particular, we included data on mean annual temperature, annual precipitation, temperature seasonality, precipitation seasonality and precipitation of the driest quarter, as these represent the main climatic gradients influencing many plant reproductive traits (Zhao *et al.*, 2018; Dorji *et al.*, 2020; Huang *et al.*, 2021). We calculated two additional climatic metrics that are closely related to plant physiology and distribution (Title and Bemmels, 2018): growing degree days, which is the sum of mean monthly temperatures for months with a mean temperature >5 °C multiplied by the number of days; and the climatic moisture index, a metric of relative wetness and aridity representing the difference between annual precipitation and potential evapotranspiration. Besides climatic variables, data on soil nutrient availability and rooting conditions were extracted from the Harmonized World Soil Database version 1.2 (Fischer *et al.*, 2008). This database provides globally harmonized information on key soil properties, such as organic carbon content, cation exchange capacity (critical for assessing nutrient availability), soil texture, bulk density, coarse fragments and soil phases (influencing root penetration, soil depth and soil volume). These factors play a fundamental role in shaping plant reproductive traits by influencing resource acquisition, water retention and root development, ultimately affecting plant fitness and adaptation across diverse environments (Lehmann and Kleber, 2015; Wieder *et al.*, 2015; Batjes, 2016).

Each species record was also matched with a potential number of pollinators. We used the relative species richness map of bees (Anthophila) (Orr *et al.*, 2021) to extract information on the potential number of pollinator species dwelling in a given spot. All abiotic and biotic characteristics were combined in a matrix of predictor variables. We screened the matrix for pairwise correlations and iteratively removed redundant variables from the dataset. In each pair of variables showing an absolute correlation *r* > 0.65, the more informative variable was retained until all pairwise correlations were less than the threshold. The subset used in the analyses involved altitude, temperature seasonality, precipitation seasonality, climatic moisture index, nutrient availability, rooting conditions and number of pollinators.

### Mating system, ploidy level and lifespan data

Mating system classifications, either self-fertilization or outcrossing, were compiled from the literature and the FloraWeb database (German Federal Agency for Nature Conservation [BfN]; www.floraweb.de), with full details provided in the Supplementary Data Dataset S1. We considered the categories ‘obligate selfer’ and ‘facultative selfer’ as selfing and ‘obligate outcrosser’ and ‘facultative outcrosser’ as outcrossing. Ploidy levels (coded as diploid and polyploid) were sourced from the BrassiBase database (Koch *et al.*, 2012, 2018; Kiefer *et al.*, 2014; https://brassibase.cos.uni-heidelberg.de/bb2/). Species classified as diploid or mesopolyploid were coded as diploids, whereas mesoneopolyploids and neopolyploids were coded as polyploids (Lysak and Koch, 2011). Additional ploidy data were obtained from the literature that estimated ploidy levels using the Stebbins fraction, a metric reflecting reproductive strategies (Román-Palacios *et al.*, 2020). Moreover, the lifespan (annual, biennial or perennial) information was extracted from BrassiBase.

### Phylogenetic reconstruction

To investigate the evolutionary history of traits, we used a species-level dataset comprising pollen number and ovule number (see above). For our phylogenetic comparative analyses, we employed a 1004-species tree of the Brassicaceae, including eight outgroup taxa from the order Brassicales. Taxonomic treatment followed BrassiBase version 2.0.1 (https://brassibase.cos.uni-heidelberg.de/bb2/). We compiled sequences of the internal transcribed spacers (ITS1 and ITS2) of nuclear ribosomal DNA, including the 5.8S gene, from BrassiBase and added sequences from the NCBI nucleotide database to increase our taxonomic sampling. We also collected sequences of the plastid *trn*L/*trn*F region and complete plastome sequences from the NCBI nucleotide database. ITS1, 5.8S and ITS2 were aligned with Muscle5 (Edgar, 2022). For the alignment of the *trn*L/*trn*F region and complete plastome sequences, we used MAFFT version 7 (Katoh and Standley, 2013) to facilitate the alignment of the long plastome sequences. The alignments were trimmed using trimAL v1.4.rev15 (Capella-Gutiérrez *et al.*, 2009), removing positions with gaps if ≥30 % of the accessions showed the gap. Additionally, a few adjustments were made manually in AliView v1.30 (Larsson, 2014). In particular, in a few cases, unalignable sequence ends bordering indels were removed. Trimming completely removed the *trn*L–*trn*F intergenic spacer, which comprised pseudogenes (Koch *et al.*, 2005). To find the best substitution model, we used ModelFinder for both regions separately (Kalyaanamoorthy *et al.*, 2017). Then, the two alignments were concatenated, and a maximum likelihood (ML) tree was constructed using IQ-TREE MPI multicore v2.3.5.cmaple (Chernomor *et al.*, 2016; Minh *et al.*, 2020), inferring branch support using 1000 ultrafast bootstrapping (Hoang *et al.*, 2018). The trimmed alignment for phylogenetic reconstruction and the ML tree are available on OSF (Haghighatnia *et al.*, 2025). This tree was made ultrametric using penalized likelihood in treePL v1.0 (Smith and O’Meara, 2012) using one fossil (*Dressiantha bicarpellata* [93.6–89.3 million years ago (Ma)]; Gandolfo *et al.*, 1998) and three secondary calibration points (crown Brassicaceae [25.7–23.1 Ma], crown lineage III [15.42–13.5 Ma] and crown *Arabidopsis* genus [6.84–5.54 Ma]; 95 % confidence intervals of the highest posterior density (HPD) values previously estimated for the respective crown ages are given; Hendriks *et al.*, 2023). Molecular dating analyses were then conducted by integrating this primarily dated phylogeny as the starting tree, the *D. bicarpellata* fossil used to calibrate the stem node of the order Brassicales, and secondary calibration points of the crown Brassicaceae and lineage III under an optimized relaxed clock rate (ORC) in BEAST v2.7.7 (Bouckaert *et al.*, 2019; Müller and Bouckaert, 2020; Douglas *et al.*, 2021). The XML file of the run is available on OSF (Haghighatnia *et al.*, 2025). Hereafter, we refer to this dated phylogeny as the main tree.

In parallel, we generated an alternative dated phylogeny incorporating genus-level relationships from the most recent Brassicaceae phylogeny reconstructed by target enrichment of 1081 nuclear genes (Hendriks *et al.*, 2023). To do this, we matched species names between the Hendriks *et al.* (2023) phylogeny and our ITS-*trn*L/*trn*F phylogeny, and pruned the tree to retain only genera with at least one shared species. This resulted in a genus-level backbone phylogeny comprising 198 species. We then reconstructed an ML phylogeny using IQ-TREE, applying the same substitution models, partition scheme and branch support estimation framework as in our primary analysis. The constrained topology argument (-g) was used to fix genus-level relationships (based on the pruned tree) while allowing phylogenetic placement of the remaining taxa to be optimized across the full dataset. This constrained tree was subsequently made ultrametric using the same penalized likelihood approach and fossil and secondary calibration points as before. We then performed molecular dating analyses in BEAST, maintaining the same criteria described previously. To preserve the genus-level relationships enforced during tree inference, we incorporated a multi-monophyletic constraint distribution in the BEAST configuration file. This ensured that all genera defined in the constrained topology were treated as monophyletic during tree sampling, while allowing species to be freely placed within their respective constrained genera. Hereafter, we refer to this dated phylogeny as the constrained tree.

To run subsequent analyses, species absent from the dataset were pruned from the time-calibrated trees using the function drop.tip in the ape package v5.8 (Paradis and Schliep, 2019) in R to exclusively retain species in the phylogeny for which trait data were available (158 species).

### Phylogenetic signal and trait evolution

Pollen and ovule numbers were log-transformed (log(*x* + 1)) for all analyses to address potential skewness and heteroscedasticity and ensure the values remained positive. The phylogenetic signal was estimated using Blomberg’s *K* (Blomberg *et al.*, 2003) and Pagel’s *λ* (Pagel, 1999) with the function phylosig implemented in the phytools package v2.3-0 (Revell, 2024) in R. To determine which continuous trait model best fits our datasets, we used the Geiger package v2.0.11 (Harmon *et al.*, 2008) in R. Three continuous trait models were tested: Brownian motion (BM), Ornstein–Uhlenbeck (OU) and early burst (EB). The BM model assumes that traits evolve randomly over time without constraints, representing neutral drift (Felsenstein, 1985). The OU process can be visualized as particles under BM experiencing friction. It assumes traits evolve towards an optimal value (adaptive peak), capturing selection signatures (Hansen, 1997). The EB model represents evolutionary scenarios where the rate of trait evolution is highest near the tree’s root and decreases over time. This model is associated with adaptive radiation, where traits diversify rapidly early in the evolutionary history before stabilizing (Harmon *et al.*, 2010). Once the best-fit model was identified, we estimated the corresponding parameters (e.g. *σ*^2^, z_0 and others) based on the chosen model to better understand the traits’ evolutionary dynamics. Ancestral state reconstructions were performed using ML estimation (Harmon, 2019). ML estimations with variances and 95 % confidence intervals were conducted using the function fastAnc in the phytools package. Ancestral state mapping was accomplished by interpolating the estimated states along each edge using Felsenstein’s equation (2) (Felsenstein, 1985). The reconstructed ancestral states were visualized on the tree using the function contMap.

### Phylogenetically independent contrasts

Phylogenetically independent contrasts (PICs) were performed following the method of Felsenstein (1985) using the function pic in the ape package v5.8. Log-transformed values of ovule and pollen numbers (log(*x* + 1)) were used to address skewness and ensure non-negative values. PICs were calculated separately for ovule and pollen numbers based on the BM model of evolution, representing independent differences between sister taxa scaled by branch lengths. A linear regression model was used to examine the relationship between the PIC-transformed pollen and ovule values, with pollen as the dependent variable. Pearson correlation was also calculated using the function cor.test to assess the phylogenetically adjusted relationship. A scatterplot was created using the ggplot2 package (Wickham, 2016), showing the PIC-transformed values, a regression line with 95 % confidence intervals, and the correlation coefficient (*r*) and *P*-value.

### Statistical analysis of mating system and ploidy level

We analysed species with available data on these traits to determine whether ploidy level and mating system explain pollen and ovule number variation. Ploidy was categorized as diploid or polyploid, while mating systems were classified either as selfing or outcrossing. Due to the lower sample availability, species with a ‘mixed’ mating system were excluded (three species). We conducted a Type II analysis of variance (ANOVA) using the function Anova from the car package v3.1-3 (Fox and Weisberg, 2018) separately for the ploidy level and mating system to assess their independent effects on pollen and ovule number. This approach accounts for potential imbalances in sample sizes and provides more robust estimates of factor significance (Langsrud, 2003). By this method, we evaluated whether either factor significantly influenced trait variation while ensuring a more reliable comparison between groups. Further, we investigated whether ploidy level and mating system interact in shaping pollen and ovule numbers, using a two-way Type II ANOVA from the car package. To compare the relative contributions of ploidy and mating system to variation in pollen and ovule count, we computed *η*² (*η*² partial) as a measure of effect size using the effectsize package (Ben-Shachar *et al.*, 2020). This metric quantifies the proportion of variance explained by each predictor, with higher values indicating a stronger effect.

To account for the impact of ploidy level and mating system on ovule and pollen numbers while accounting for phylogenetic non-independence, we performed phylogenetic generalized least squares (PGLS) regression. We first created a correlation structure using the corMartins function in ape package v5.8. PGLS models were implemented using the function gls from the nlme package v3.1-164 (Pinheiro, 2011). Pollen and ovule numbers were assigned as dependent variables, while ploidy and mating systems were categorical predictors. To determine whether phylogenetic correlation was necessary, we also fitted the same models using ordinary least squares (OLS) via the function lm from the stats package, which assumes species observations are independent. To compare PGLS and OLS, we computed Akaike information criterion (AIC) values for each model, where lower AIC values indicate better model fit.

### Statistical analysis of environmental factors (abiotic and biotic)

We employed generalized additive mixed models (GAMMs) (Wood, 2014) to assess how pollinator availability and abiotic factors influence pollen and ovule production. Pollen and ovule numbers were modelled as Poisson-distributed responses with a logarithmic link function. Since the full models showed considerable overdispersion (*ϕ* ≫ 1), both GAMMs were refitted with observation-level random effects to account for unmodelled heterogeneity (Harrison, 2014). GAMMs were fitted using the gamm4 package (Wood, 2014), incorporating thin plate regression spline smooths (Wood, 2003) of preselected environmental predictors. Given our hierarchical sampling design, with multiple observations per individual nested within species, genera and tribes, we accounted for non-independence in observations by including a nested phylogenetic random effect structure. To build the most parsimonious models, coefficients of variables with weak predictive performance were shrunk towards zero using double-penalty smoothing (Marra and Wood, 2011). Model diagnostics were assessed using residual plots, and no significant violations of the model assumptions were detected. We evaluated model fit using *R*^2^ and adjusted intraclass correlation coefficients (ICCs) following Nakagawa *et al.* (2017). Data visualization was conducted using ggplot2 (Wickham, 2016), and model performance metrics were assessed using the performance package (Lüdecke *et al.*, 2021).

All analyses using different packages were performed in R v4.3.3 (R Core Team, 2023). AI assistant (GPT-4o, OpenAI) was used to debug R scripts, enhance code efficiency, modify scripts to improve figure aesthetics and select colour-blind-friendly palettes. It also aided in tuning BEAST2 XML operator settings to improve MCMC mixing and reduce setup time. The explanation of the prompt that helped to narrow down the number of operators to modify for the run is available on OSF (Haghighatnia *et al.*, 2025).

## RESULTS

### Strong phylogenetic signal for ovule number compared with pollen number

Phylogenetic reconstruction inherently varies based on methodological and marker selection, potentially influencing the inferred evolutionary trajectories. Our main tree, estimated within a Bayesian framework, is based on the classical marker set of nuclear ITS and plastid *trn*L/*trn*F due to the unavailability of phylogenomic data for the majority of the species used in this study. Notably, the recent phylogenetic tree by Hendriks *et al.* (2023) included only 60 of the 158 species for which we had trait data, necessitating a *de novo* reconstruction to ensure full trait–phylogeny alignment. Node support in the main tree was moderate, with an average posterior probability of 0.78 (range 0–1) for backbone nodes and 0.75 for shallower nodes. At the tribal level, the average posterior probability was 0.77, indicating that the node support is consistent across the tree. To complement this reconstruction and incorporate robust genus-level relationships, we also built a constrained tree using the tree topology by Hendriks *et al.* (2023) to enforce monophyly of genera while allowing species-level placements to vary freely within each genus.

Pollen and ovule production showed a substantial variation among the 158 studied Brassicaceae species. Pollen numbers ranged from 22 to 234 228 grains, with a mean (± standard deviation) of 36 007 ± 41 201 grains and a high coefficient of variation of 114 %. Ovule number showed lower variability, ranging from 1 to 153, with a mean of 25 ± 26 and a coefficient of variation of 104 %.

We then aimed to determine the role of mere phylogeny in the interspecific variation for pollen and ovule numbers. Pagel’s *λ* values indicated a strong phylogenetic signal for ovule number (*λ* = 0.95) and a moderate signal for pollen number (*λ* = 0.74) in the main tree. The constrained tree yielded similar results (*λ* = 0.94 for ovule number and *λ* = 0.75 for pollen number), suggesting that ovule number is more evolutionarily constrained by lineage history. This pattern was consistent with Blomberg’s *K* estimates, with 0.44 for pollen and 0.61 for ovule number in the main tree, and 0.52 and 0.66, respectively, in the constrained tree. The significance of these phylogenetic signals was confirmed through 1000 randomizations (*P* = 0.001), reinforcing that while both traits exhibit phylogenetic structure, ovule number is more strongly conserved across evolutionary lineages than pollen number in both trees. A likelihood ratio (LR) test comparing models with estimated *λ* with models assuming no phylogenetic signal (*λ* = 0) confirmed the presence of a robust phylogenetic structure. For the main tree, the LR statistic was 57.95 for pollen number (*P* = 2.68 × 10^−14^) and 89.60 for ovule number (*P* = 2.9 × 10^−21^). Results from the constrained tree were consistent, with LR = 60.4 (*P* = 7.8 × 10^−15^) for pollen and LR = 85 (*P* = 3.02 × 10^−20^) for ovule number. These results further confirm the presence of a robust phylogenetic structure in the data (Supplementary Data Tables S1 and S2). However, the relatively moderate *K* values for pollen and ovule numbers suggest that a substantial fraction of the observed variation deviates from the BM model’s expectations, potentially reflecting the influence of adaptive evolution, environmental heterogeneity or other non-phylogenetic factors.

### Adaptive evolution explains the evolution of ovule and pollen numbers in the Brassicaceae

**Fig. 1.**
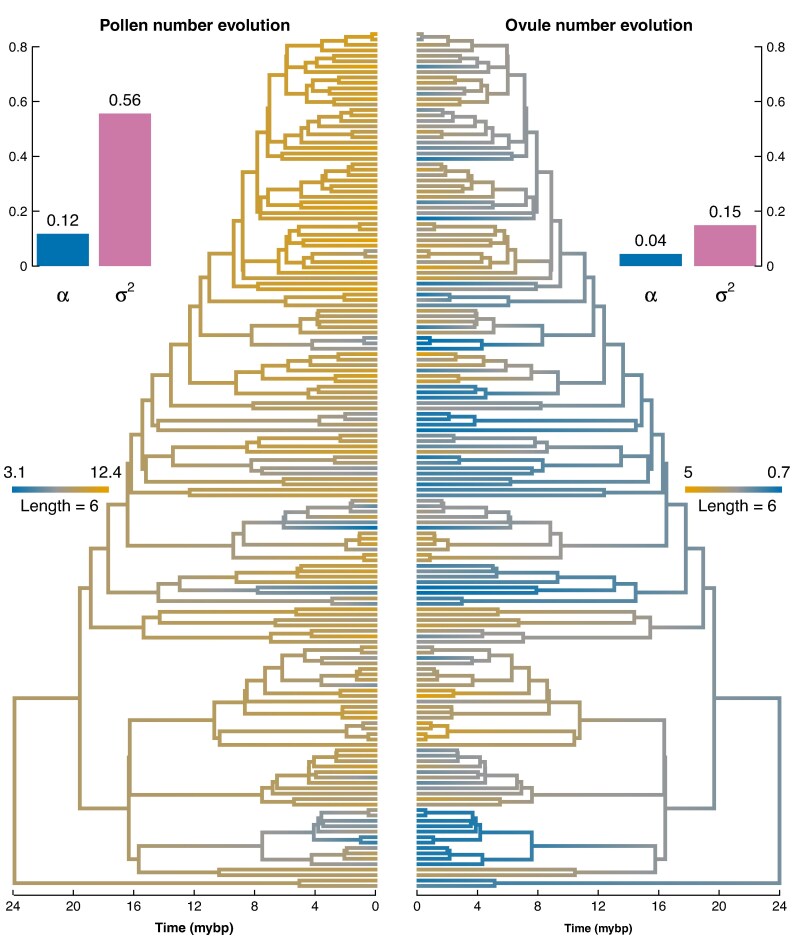
Evolutionary patterns of pollen and ovule numbers in the Brassicaceae based on the main tree. Continuous trait maps depicting the evolution of (left) log-transformed pollen number and (right) log-transformed ovule number. Trait values are represented using a gradient that transitions from blue (low values) through grey (medium values) and gold (high values). Time is scaled in millions of years before the present (mybp). Bar plots adjacent to each phylogeny display the estimated parameters from the best-fitting Ornstein–Uhlenbeck model: the strength of selection (*α*) and evolutionary variance (*σ*²) for pollen and ovule numbers.

The evolution of ovule and pollen numbers in the Brassicaceae was analysed using comparative evolutionary models, with the OU model emerging as the best fit for both traits and both trees (Supplementary Data Tables S3 and S4). This conclusion is supported by the lowest AIC values and highest AIC weights among the tested models. For ovule number, the AIC values for the BM, EB and OU models were 406.8, 408.8 and 403.9, respectively, in the main tree. Using the constrained tree, the corresponding AIC values were slightly higher: 409.13 (BM), 411.13 (EB) and 406.39 (OU). The OU model showed the strongest support with an AIC weight of 0.76, compared with 0.18 for BM and 0.07 for EB in the main tree. The constrained tree yielded slightly lower support for the OU model but retained it as the best fit, with AIC weights of 0.74 (OU), 0.19 (BM) and 0.07 (EB). While the preference for the OU model indicates that ovule number evolution is primarily driven by stabilizing selection towards an adaptive optimum, the moderate support for the BM model suggests that random drift also shapes variation. Parameter estimates for the OU model revealed a relatively low rate of adaptation (*α* = 0.04 for both trees) and a low rate of evolutionary variance (*σ*² = 0.15 for the main tree; 0.14 for the constrained tree) (Supplementary Data Tables S3 and S4), indicating a slow evolutionary process predominantly shaped by stabilizing selection (Fig. 1 and Supplementary Data Fig. S2, right).

For pollen number, the AIC values for the BM, EB and OU models were 568.7, 570.7 and 543.8, respectively, in the main tree. Using the constrained tree, the corresponding values were 557.90 (BM), 560.06 (EB) and 538.28 (OU). In both cases, the OU model showed a high support, with an AIC weight of nearly 1. In contrast, the BM and EB models had negligible support, with AIC weights approaching zero in both trees (Supplementary Data Tables S3 and S4). The dominance of the OU model for pollen number suggests that its evolution is driven almost entirely by stabilizing selection, with minimal contribution from stochastic processes. The OU model parameters for pollen number showed a higher rate of adaptation (*α* = 0.12 for the main tree; 0.10 for the constrained tree) and greater evolutionary variance (*σ*² = 0.56 for main tree; 0.46 for the constrained tree) compared with ovule number, reflecting a faster evolutionary process with a larger variation (Fig. 1 and Supplementary Data Fig. S2, left). These findings indicate that both traits are shaped by stabilizing selection, while their evolutionary dynamics differ. This difference in evolutionary dynamics between pollen and ovule numbers was also supported by PICs, which revealed a statistically significant but weak positive correlation between pollen and ovule numbers across the Brassicaceae (Supplementary Data Figs S3 and S4). The Pearson correlation coefficient for the PIC-transformed values in the main tree was *r* = 0.21 (*P* = 0.009), and *r* = 0.22 for the constrained tree (*P* = 0.0067), indicating a modest but statistically significant evolutionary association between these traits after accounting for phylogenetic relatedness.

### Environmental conditions are weakly associated with pollen and ovule number evolution

**Fig. 2.**
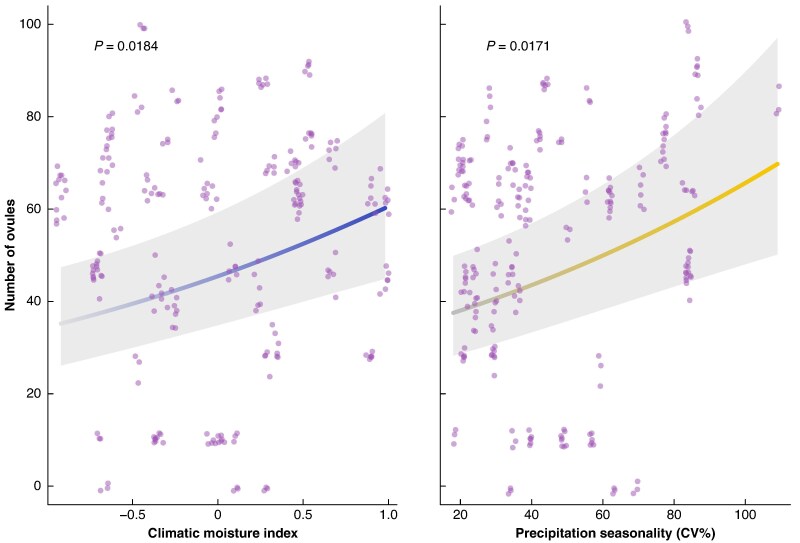
Significant relationships between number of ovules, climatic moisture index (left) and precipitation seasonality (right). Model predictions (lines) and their 95 % confidence intervals (grey bands) are displayed. Note that negative values of the climatic moisture index indicate moisture deficits, values close to zero (light grey) represent a balance between moisture supply and demand, while positive values indicate wetter conditions (blues). For precipitation seasonality, grey and gold are representative of stable and extreme seasonality, respectively. The points represent raw measured values for each individual, ranging up to ∼100 ovules. Points are slightly jittered in both horizontal and vertical directions to reduce overlap and improve visual clarity.

We then investigated the role of ecological conditions in shaping the evolution of pollen and ovule numbers. For this purpose, we measured pollen and ovule numbers in a subset of species cultivated in a common garden (see Materials and methods, Supplementary Data Dataset S2) testing associations between environmental conditions of origin and pollen or ovule number. The environmental association with ovule number was relatively weak (*R*^2^ = 3.3 %), while the phylogenetic signal had the strongest impact. Genus played the most pronounced role (ICC = 67.0 %) followed by species (22.5 %) and tribe (10.5 %). Regarding the influence of the native environment, the most parsimonious model involved a significant impact of precipitation seasonality (GAMM: effective degrees of freedom [e.d.f.] = 0.84, *χ*^2^ = 4.57, *P* = 0.0171) and climatic moisture index (e.d.f. = 0.83.0, *χ*^2^ = 4.54, *P* = 0.0184). Other environmental characteristics showed no significant association (Supplementary Data Table S5). Both variables were positively associated with ovule number (Fig. 2), implying that plants in moist environments with higher seasonality evolved to produce more ovules.

**Fig. 3.**
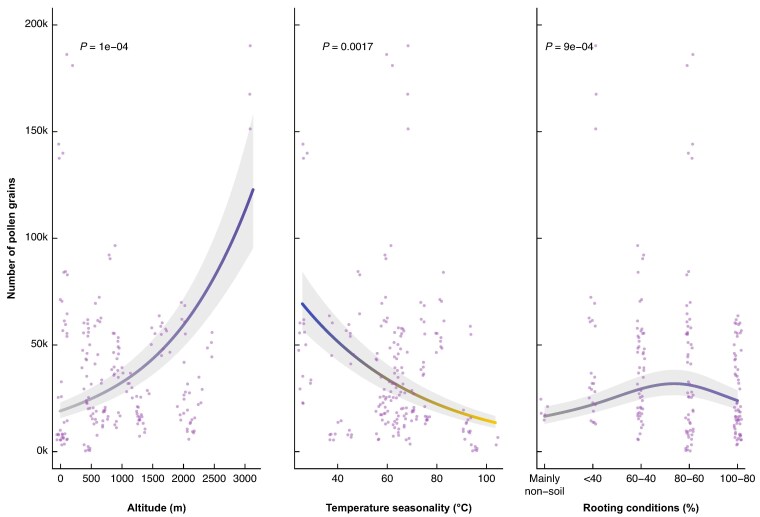
Significant relationships between the number of pollen grains, altitude (left), temperature seasonality (middle) and rooting conditions (right). Model predictions (lines) and their 95 % confidence intervals (grey bands) are displayed. Rooting conditions represent the percentage of plant growth potential. The points represent raw measured values for each individual, ranging up to ∼188 000 grains. Points were slightly jittered in both horizontal and vertical directions to reduce overlap and enhance the visual distinction of individual observations.

The environmental association with pollen number was comparable to that for ovules (*R*^2^ = 3.8 %), and the parsimonious GAMM involved significant effects of altitude (e.d.f. = 1.06, *χ*^2^ = 22.3, *P* < 0.0001), temperature seasonality (e.d.f. = 0.93, *χ*^2^ = 8.44, *P* = 0.0017) and rooting conditions (e.d.f. = 1.73, *χ*^2^ = 11.7, *P* = 0.0009) (Supplementary Data Table S5). The highest number of pollen grains is expected in conditions of high altitude, low-temperature seasonality and favourable rooting conditions (Fig. 3). The phylogenetic signal was strong, with tribe and species accounting for the most variability in pollen number (ICC = 17.7 and 76.2 %, respectively), while genus variation was less important (<0.1 %).

For both traits, no significant association was found with lifespan (annual vs perennial) and pollinator richness (Supplementary Data Table S5).

### Mating system drives the evolution of pollen production but does not affect ovule number variation

**Fig. 4.**
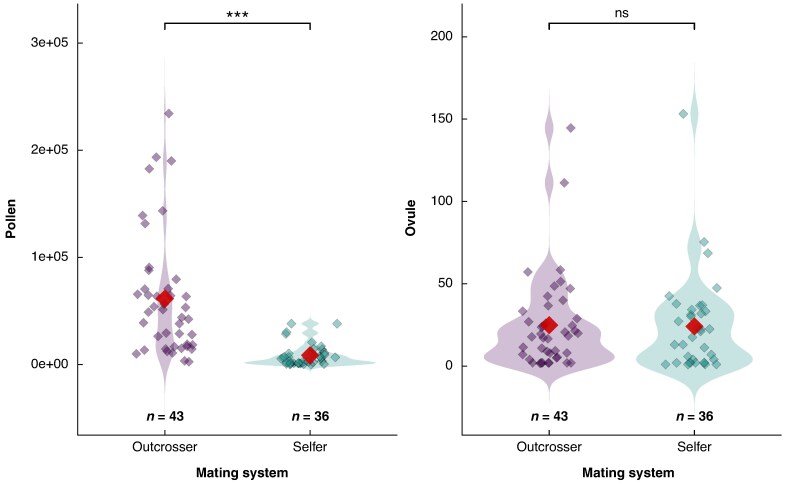
Effects of the mating system on pollen and ovule production in the Brassicaceae. Violin plots with individual data points showing the distribution of (left) log-transformed pollen numbers and (right) ovule numbers in species with different mating systems: outcrossers and selfers. Red diamonds show the mean values of pollen and ovule numbers in different mating systems. Pollen numbers are significantly higher in outcrossers compared with selfers (Type II ANOVA, ***P = 0.001), indicating a strong influence of the mating system on male reproductive investment. In contrast, ovule numbers do not differ significantly between mating systems (ns, not significant), suggesting ovule production is independent of mating strategy.

Since phylogeny had a moderate impact on pollen and ovule number evolution, we investigated the role of species traits such as mating system and ploidy level as evolutionary drivers of these traits. In the two-way ANOVA, when both ploidy and the mating system were analysed together, the mating system was the primary factor influencing pollen production (*F*_1,70_ = 24.11, *P* = 0.0001, *η*² = 0.26), with selfing species producing significantly fewer pollen grains than outcrossers (Fig. 4). Ploidy did not have a significant effect (*F*_1,70_ = 2.73, *P* = 0.10, *η*² = 0.04) (Supplementary Data Table S6). For ovule count, neither the mating system (*F*_1,70_ = 0.0002, *P* = 0.98, *η*² = 0) nor ploidy (*F*_1,70_ = 0.1728, *P* = 0.68, *η*² = 0.002) showed a significant effect. The interaction between ploidy and mating system was not significant for either trait, affecting neither pollen (*F*_1,70_ = 0.91, *P* = 0.34, *η*² = 0.01) nor ovule number (*F*_1,70_ = 0.77, *P* = 0.383, *η*² = 0.01) (Supplementary Data Table S6).

The PGLS analysis, accounting for phylogenetic correlation, revealed that phylogenetic correction improved model fit, with the PGLS model (AIC = 1789.12 for the main tree, 1792.34 for the constrained tree) performing better than the OLS model (AIC = 1793.99) (Supplementary Data Tables S7 and S8). For the main tree, the mating system was a significant predictor of pollen number (PGLS, *β* = 43,006.68, s.e. = 14,351.05, *t* = 3.00, *P* = 0.0038; OLS, *P* = 0.001), with outcrossers producing significantly more pollen than selfers. In contrast, ploidy (*β* = 7349.18, s.e. = 12 463.33, *P* = 0.557) and the interaction between ploidy and mating system (*β* = −14 636.33, s.e. = 17 612.06, *P* = 0.409) were not significant. The residual standard error was higher in the PGLS model (57 216.07) than the OLS model (42 740), suggesting that, while phylogenetic correction accounts for evolutionary relatedness, considerable variability in pollen number remains, likely influenced by additional factors. For ovule number, the PGLS model (AIC = 705.19) outperformed the OLS model (AIC = 716.59), indicating that phylogenetic correction improved model fit. None of the tested predictors were significant: ploidy (*β* = 5.27, s.e. = 8.22, *t* = 0.61, *P* = 0.524), mating system (*β* = −3.47, s.e. = 9.47, *t* = −0.37, *P* = 0.715) or their interaction (*β* = 1.04, s.e. = 11.62, *t* = 0.09, *P* = 0.929) (Supplementary Data Table S7). The constrained tree yielded similar results for both traits (Supplementary Data Table S8). This suggests that ovule number is largely conserved across evolutionary lineages, shaped more by historical constraints than by recent reproductive shifts or whole genome duplication. In contrast, pollen number is more evolutionarily flexible and strongly influenced by the mating system.

## DISCUSSION

Reproductive investment strategies are crucial for plant fitness, influencing mating success, fecundity and adaptation. Pollen and ovule production, as key reproductive traits, vary widely across species, yet the relative influence of evolutionary constraints, environmental adaptation and reproductive strategies in shaping these traits remains unresolved. This study addresses this question, examining the phylogenetic constraint of pollen and ovule production, how abiotic factors (e.g. moisture, altitude) and biotic factors (pollinator availability) influence both traits, and the extent to which mating system shifts and ploidy affect their evolution.

Using phylogenetic comparative analyses of 158 Brassicaceae species and common garden cultivation to control for phenotypic plasticity, we found that ovule number is strongly constrained by phylogeny, while pollen number is more evolutionarily flexible, largely due to mating system shifts, selfing species producing significantly less pollen. The associations between environment and both traits were limited yet significant. Ovule number increased in wetter environments, while pollen number was influenced by altitude and temperature seasonality. Whole-genome duplication had no significant impact on the evolution of either trait.

### Distinct evolutionary patterns for pollen and ovule production

Understanding evolutionary rate variation helps explain how morphological diversity arises and persists (Foote, 1997; Sidlauskas, 2008). Traits evolve at different rates; some change rapidly under strong selection, while others are constrained by development, functional trade-offs or phylogenetic inertia (Pagel, 1999; O’Meara *et al.*, 2006; Cooper and Purvis, 2010; Uyeda *et al.*, 2011). Our results show distinct selective pressures on pollen and ovule numbers: ovule number was more conserved, while pollen number was more flexible across the phylogeny (Fig. 1 and Supplementary Data Fig. S2). This pattern is consistent with other families like Lamiaceae and Boraginaceae exhibiting deep phylogenetic conservatism in ovule number, while others, such as Rosaceae, show greater variation (Potter *et al.*, 2007; Burd *et al.*, 2009). The strong phylogenetic signal observed in our study (Blomberg’s *K* = 0.61, Pagel’s *λ* = 0.95 for the main tree; 0.66 and 0.94, respectively, for the constrained tree) and the weak association with environmental factors (*R*^2^ = 3.3 %) suggest that ovule number is primarily constrained by historical lineage-specific factors in Brassicaceae rather than being highly responsive to other factors, such as ecology. Ovule production was also unaffected by mating system shifts and ploidy. Stabilizing selection models predict that traits crucial to fitness remain within an optimal range to minimize deleterious variation (Waddington, 1942; Debat and David, 2001; Flatt, 2005). Our findings align with this framework, with the OU model indicating that ovule number evolves under strong selective constraints, reflected in its slow evolutionary rate (*α* = 0.04, *σ*² = 0.15 for the main tree; 0.04 and 0.14, respectively, for the constrained tree).

A likely explanation for this constraint lies in the fundamental trade-off between reproductive output and offspring survival. Maternal resources are finite, and plants must allocate them strategically to balance ovule production with seed provisioning. Producing more ovules can come at the cost of per-seed investment, potentially reducing overall reproductive success (Haig and Westoby, 1988; Greenway and Harder, 2007). This limitation can impose an evolutionary restriction, as excessive ovule production without sufficient resource provisioning could reduce seedling fitness and long-term reproductive success. With this in mind, whether investment per seed is fully correlated to ovule number remains to be investigated, and it is likely that the evolution of maternal investment follows its own trajectory to a certain extent. Genetic constraints may also limit the evolutionary flexibility of ovule number, as developmental regulation often restricts trait variability (Hallgrímsson *et al.*, 2009). In *Arabidopsis thaliana*, genes involved in ovule development also play essential roles in broader reproductive processes, including gametophyte formation and carpel development, with mutations often leading to reduced fertility and structural defects (Pinyopich *et al.*, 2003). Moreover, ovule number and gynoecium size are interconnected traits governed by shared hormonal and genetic pathways, meaning that changes in ovule production can have cascading effects on floral architecture and seed yield (Cucinotta *et al.*, 2020). Because of these pleiotropic effects, strong selection likely acts to maintain ovule number within an optimal range, ensuring reproductive success while preserving developmental stability.

Compared with ovule production, pollen number was more evolutionarily flexible, exhibiting a moderate phylogenetic signal (Blomberg’s *K* = 0.44, Pagel’s *λ* = 0.74 for the main tree; 0.52 and 0.75, respectively, for the constrained tree) and higher evolutionary rate (*α* = 0.12, *σ*² = 0.56 for the main tree; 0.10 and 0.46, respectively, for the constrained tree). This pattern contrasts previous studies reporting strong phylogenetic constraints for pollen number in several plant communities (Arroyo *et al.*, 2019; Nepal *et al.*, 2023). This evolutionary flexibility largely arises from the well-known influence of mating systems on pollen production (Cruden, 1977; Tsuchimatsu and Fujii, 2022). Specifically, selfing species produced significantly fewer pollen grains than outcrossers (Fig. 4), confirmed by phylogenetically corrected PGLS, indicating that reproductive strategies are major drivers of pollen number evolution. The different impact of mating system shifts on ovule and pollen production, similarly found in other species (Harder and Johnson, 2023), may reflect the different factors limiting the reproductive success of male and female functions – mating opportunities and maternal resources, respectively, according to Bateman’s principle. This is consistent with the idea that outcrossing species face higher competition among pollen grains for fertilization success, favouring increased pollen output to maximize pollination opportunities and reproductive success (Cruden, 1977; Harder and Johnson, 2008). In contrast, selfing species experience reduced pollen competition and guaranteed fertilization, allowing selection to favour lower pollen production as a resource-conserving strategy (Barrett, 2002; Sicard and Lenhard, 2011). Another factor potentially explaining the relative evolutionary flexibility of pollen production compared with ovule production might be different genetic constraints. Indeed, the effect of genes influencing pollen production might be more modular and less restrictive than ovule-regulating genes. The *REDUCED POLLEN NUMBER1* (*RDP1*) gene exemplifies this flexibility; mutations in this gene reduce pollen number but do not seem to influence other traits, highlighting weaker pleiotropic constraints (Kakui *et al.*, 2022). This is the case of one gene, however, and whether the genetic architecture underlying pollen production follows this trend remains to be explored.

Although both traits are influenced by phylogenetic factors, their evolutionary paths are largely independent, as suggested by the weak but significant phylogenetic correlation (*r* = 0.21, *P* = 0.009 for the main tree; 0.22 and 0.0067, respectively, for the constrained tree) between pollen and ovule numbers (Supplementary Data Figs S2 and S3). Besides mating systems, the differing environmental associations with ovule and pollen numbers (see next section) further support the idea that these traits follow distinct evolutionary pathways. Together, these patterns suggest that while both traits respond to phylogenetic and ecological factors, they do so through largely independent mechanisms, shaped by different environmental constraints and adaptive pressures.

### Is pollen production ‘cheap’?

Pollen is often viewed as a low-cost male investment due to its small size and excess production compared with ovules (Charnov, 1982; Harder and Routley, 2006). However, this overlooks ecological and evolutionary factors influencing pollen production. Hermaphroditic plant species must balance resource allocation between pollen and ovules, following resource allocation theory, which predicts optimal investment to maximize fitness under constraints (Charnov, 1982; Lloyd, 1984; Obeso, 2002). Pollen costs depend on quantity, viability, dispersal efficiency and gamete success, requiring carbon and nitrogen inputs (Nepi and Pacini, 1993; Ashman and Schoen, 1994; Delph and Ashman, 2006; Franchi *et al.*, 2011). The assumption that pollen is inherently ‘cheap’ is largely based on Bateman’s principle, which itself is an assumption based on the relative number and size of pollen grains versus ovules rather than direct cost comparison (Cruden, 1977; Charnov, 1982).

To examine this assumption, we tested how pollen and ovule numbers evolve in response to selective pressures. The association between environmental factors and both traits was weak and similar, with ovule number showing an *R*^2^ of 3.3 % and pollen number an *R*^2^ of 3.8 %. Ovule production appeared primarily shaped by water availability, as indicated by its association with precipitation seasonality and climatic moisture levels (Fig. 2). This suggests that plants may adjust ovule investment in response to water resources, potentially under resource (water) constraints. In contrast, pollen number was associated with a broader range of environmental factors, including altitude, temperature fluctuations and rooting conditions (Fig. 3). Altitude and rooting conditions also suggest resource constraints on the evolution of pollen production, to a similar extent as ovule production according to *R*^2^ values.

Beyond environmental associations, the reduction in pollen number in selfing species across Brassicaceae supports a model of resource allocation optimization (Stearns, 1998; Roff, 2002), where selection would favour lower pollen production as a resource-conserving strategy in selfers (Barrett, 2002; Sicard and Lenhard, 2011). Further supporting this, in the selfer *A. thaliana* a mutation that reduces pollen production was found to be under positive selection (Tsuchimatsu *et al.*, 2020). This indicates that rather than relaxed selection, lower pollen production confers a selective advantage, likely by reducing the reproductive cost.

Overall, these findings challenge the traditional view that pollen is a negligible investment. The evolution of pollen production did not appear less resource-constrained than for ovule production, and the consistent reduction in pollen number in selfers may be adaptive due to its significant cost reduction.

### Pollinator availability may explain the evolution of pollen production

Pollinators are vital for plant reproduction, and it has been suggested that pollen and ovule production evolve in response to pollinator fluctuations (Burd *et al.*, 2009; Ollerton, 2017; Tong *et al.*, 2023). In particular, increased ovule production in pollination-limited environments could maximize reproductive opportunities if pollination occurs, while pollen production might be more directly shaped by selection to enhance male reproductive success under pollinator scarcity (Harder and Johnson, 2023).

Our results suggest that pollinator availability is not a primary driver of pollen investment in the Brassicaceae. Despite the role of pollen production in male competition (Snow and Spira, 1991; Lankinen and Skogsmyr, 2002), its variation was not linked to pollinator richness in our study. Nevertheless, pollen production increased with altitude, supporting the hypothesis that in pollinator-scarce environments, such as higher elevations, plants may produce more pollen to counteract reduced mating opportunities (Harder and Johnson, 2023). Alternatively, a short growing season at higher altitudes may select for more pollen-rich flowers, which would maximize each visit by pollinators and thereby improve male fitness. In contrast, ovule production remained unaffected by pollinator richness or altitude, challenging the hypothesis that plants compensate for stochastic pollination by increasing ovule production (Burd *et al.*, 2009). This finding also contrasts with a study reporting higher ovule numbers at high elevations, potentially as a response to reduced pollination opportunities (Arroyo *et al.*, 2019). Overall, our results are partially consistent with empirical studies suggesting that pollen production is more responsive to pollinator availability than ovule production (Harder and Johnson, 2023).

However, one clear limitation of our study is the reliance on bee species richness as a proxy for pollinator availability (Orr *et al.*, 2021), which overlooks factors like pollinator abundance, visitation frequency, or floral fidelity – key predictors of effective pollination and plant reproductive success (Vázquez *et al.*, 2005; Klein *et al.*, 2007; Winfree *et al.*, 2009; Brosi and Briggs, 2013; Goulson *et al.*, 2015). Thus, the absence of a relationship between pollinator richness and reproductive traits may reflect the limitations of our proxy rather than a true lack of pollination effects on reproductive allocation. Similarly, altitude is only an indirect proxy for pollinator abundance. Future research using direct pollination observations and pollen deposition analyses could refine our understanding of selective pressures, challenging current evolutionary frameworks for plant reproductive strategies.

### Conclusions

Our findings reveal that reproductive trait evolution reflects phylogenetic constraints and adaptive responses. Ovule number is evolutionarily conserved, showing weak environmental association and no significant effect of mating system, suggesting strong stabilizing selection. In contrast, pollen number is more evolutionarily flexible and primarily shaped by mating system shifts rather than environmental variation. The consistent reduction in pollen production in selfing species, along with evidence for positive selection on pollen-reducing mutations in *A. thaliana*, challenges the assumption that pollen is a low-cost investment. Instead, pollen production carries a cost, which plants minimize when selection favours increased efficiency under self-fertilization. While we found no direct link between pollinator richness and reproductive investment, the observed increase in pollen production at high elevations suggests that pollen output may adjust to pollination limitations in certain environments. Given the limitations of our proxy for pollinator availability, future studies incorporating direct measures of pollination success will be crucial for refining theories on male reproductive allocation in plants.

## Supplementary Material

mcaf176_Supplementary_Data

## Data Availability

The final alignment for building the phylogeny and the maximum likelihood tree (IQ-TREE) is available online on OSF (https://doi.org/10.17605/OSF.IO/XMREJ).

## SUPPLEMENTARY DATA

Supplementary data are available at *Annals of Botany* online and consist of the following. Figure S1: geographical distribution of species based on population-level sampling. Figure S2: evolutionary patterns of pollen and ovule numbers in the Brassicaceae based on the constrained tree show similar trends to those observed in the main tree. Figure S3: phylogenetic independent contrasts (PICs) between pollen and ovule numbers show a statistically significant but weak positive correlation in the main tree. Figure S4: phylogenetically independent contrasts (PICs) between pollen and ovule numbers show a statistically significant but weak positive correlation in the constrained tree. Table S1: phylogenetic signal analysis of pollen and ovule numbers based on the main tree. Table S2: phylogenetic signal analysis of pollen and ovule numbers based on the constrained tree. Table S3: comparative phylogenetic model evolution for pollen and ovule numbers based on the main tree. Table S4: comparative phylogenetic model evolution for pollen and ovule numbers based on the constrained tree. Table S5: results of generalized additive mixed (GAM) models testing the effects of biotic and abiotic variables on pollen and ovule production. Table S6: ANOVA results and effect sizes for pollen and ovule numbers. Table S7: summary of PGLS (OU) and OLS results with AIC comparisons based on the main tree. Table S8: summary of PGLS (OU) and OLS results with AIC comparisons based on the constrained tree. Dataset S1: complete dataset of 158 Brassicaceae species, with information on the mating system and ploidy level used for all phylogenetic and trait analyses. Dataset S2: complete dataset of cultivated species with abiotic and biotic measurements, and GPS coordinates used to examine the relationship between reproductive traits and environmental factors.
