# Supplementary material for: Divergent evolutionary trajectories of male and female investment in the Brassicaceae
Source: Ann Bot. 2025 Aug 4;138(3):868–82. doi: 10.1093/aob/mcaf176 (PMC13487425; doi:10.1093/aob/mcaf176)
Supplement: Supplementary file 1 [file mcaf176_supplementary_data.zip › Fig. S1.pdf]

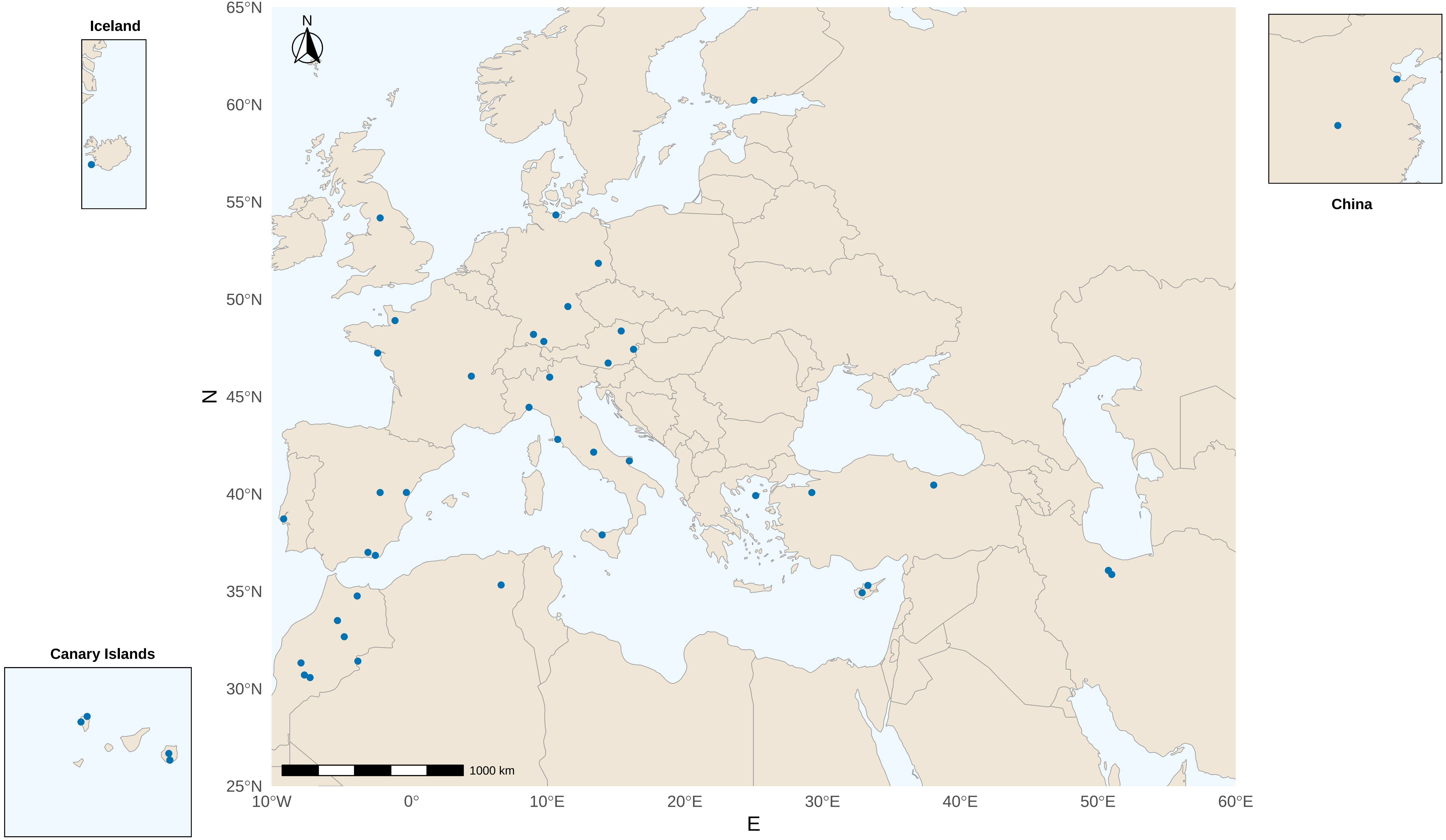
Iceland
China
Canary Islands
N
65°N
60°N
55°N
50°N
45°N
40°N
35°N
30°N
25°N
N
10°W
0°
10°E
20°E
30°E
40°E
50°E
60°E
E
1000 km
