# Supplementary material for: Divergent evolutionary trajectories of male and female investment in the Brassicaceae
Source: Ann Bot. 2025 Aug 4;138(3):868–82. doi: 10.1093/aob/mcaf176 (PMC13487425; doi:10.1093/aob/mcaf176)
Supplement: Supplementary file 1 [file mcaf176_supplementary_data.zip › Fig. S2.pdf]

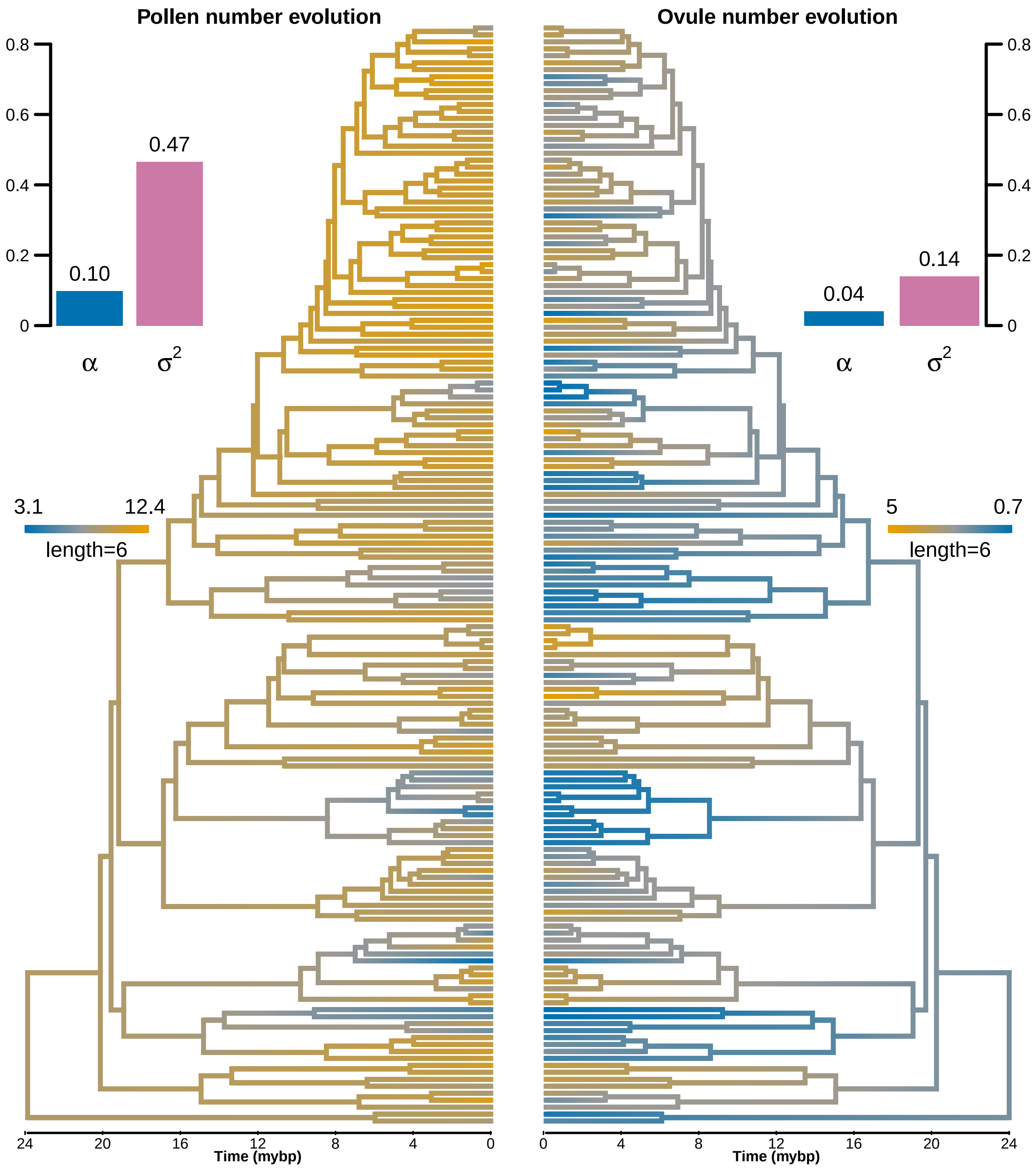
Pollen number evolution
Ovule number evolution
0.8
0.6
0.4
0.2
0
0.10
0.47
0.04
0.14
α
σ2
3.1
12.4
length=6
5
0.7
length=6
24
20
16
12
8
4
0
Time (mybp)
