# Supplementary material for: Divergent evolutionary trajectories of male and female investment in the Brassicaceae
Source: Ann Bot. 2025 Aug 4;138(3):868–82. doi: 10.1093/aob/mcaf176 (PMC13487425; doi:10.1093/aob/mcaf176)
Supplement: Supplementary file 1 [file mcaf176_supplementary_data.zip › Fig. S4.pdf]

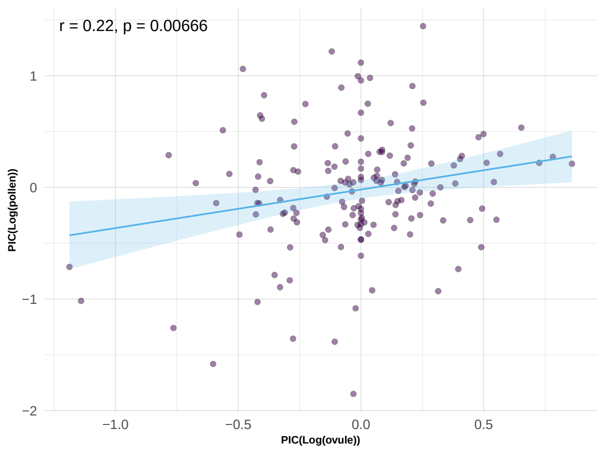

r = 0.22, p = 0.00666
PIC(Log(pollen))
PIC(Log(ovule))
1
0
−1
−2
−1.0
−0.5
0.0
0.5
