# Supplementary material for: Divergent evolutionary trajectories of male and female investment in the Brassicaceae
Source: Ann Bot. 2025 Aug 4;138(3):868–82. doi: 10.1093/aob/mcaf176 (PMC13487425; doi:10.1093/aob/mcaf176)
Supplement: Supplementary file 1 [file mcaf176_supplementary_data.zip › Revised_Supplementary_tables.docx]

**Table S1.** Phylogenetic signal analysis of pollen and ovule numbers based on the main tree. The signals are estimated using two methods: Blomberg’s K with p-value (K) and Pagel’s Lambda with logL and likelihood ratio (LR) and LR p-values. Significant p-values are shown with strike and bold font.

| Metric | Pollen | Ovule |
| --- | --- | --- |
| Blomberg’s K | 0.44 | 0.61 |
| P-value (K) | **0.001*** | **0.001*** |
| Pagel’s Lambda | 0.74 | 0.95 |
| logL (Lambda) | -268.11 | -775.07 |
| LR (Lambda=0) | 57.95 | 89.61 |
| P-value (LR Test) | **2.68E-14*** | **2.9E-21*** |

**Table S2.** Phylogenetic signal analysis of pollen and ovule numbers based on the constrained tree. The signals are estimated using two methods: Blomberg’s K with p-value (K) and Pagel’s Lambda with logL and likelihood ratio (LR) and LR p-values. Significant p-values are shown with strike and bold font.

| Metric | Pollen | Ovule |
| --- | --- | --- |
| Blomberg’s K | 0.52 | 0.66 |
| P-value (K) | **0.001*** | **0.001*** |
| Pagel’s Lambda | 0.75 | 0.94 |
| logL (Lambda) | -266.57 | -199.4 |
| LR (Lambda=0) | 60.4 | 85.00 |
| P-value (LR Test) | **7.8E-15*** | **3.02E-20*** |

**Table S3.** Model evolution of pollen and ovule numbers based on the main tree. All the models tested for their evolution are shown, including Brownian Motion (BM), Early Burst (EB), and Ornstein-Uhlenbeck (OU). Akaike information criterion (AIC), corrected AIC (AICc), AIC weight (AICw), estimated evolutionary variance (σ²), and ancestral state (z_0) are shown for all models. The strength of selection (α) is estimated for the OU model. The best-fitting models, identified by the lowest AICc values, are shown in bold.

| Model | log-likelihood | AIC | AICc | σ² | α (OU) | z_0 | AICw |
| --- | --- | --- | --- | --- | --- | --- | --- |
| BM_pollen | -282.33 | 568.66 | 568.74 | 0.31 | - | 9.26 | 0.00 |
| EB_pollen | -282.33 | 570.66 | 570.82 | 0.31 | - | 9.26 | 0.00 |
| **OU_pollen** | **-268.89** | **543.79** | **543.95** | **0.56** | **0.12** | **9.35** | **1.00** |
| BM_ovule | -201.39 | 406.77 | 406.85 | 0.11 | - | 2.37 | 0.18 |
| EB_ovule | -201.39 | 408.77 | 408.93 | 0.11 | - | 2.37 | 0.07 |
| **OU_ovule** | **-198.95** | **403.90** | **404.05** | **0.15** | **0.04** | **2.45** | **0.76** |

**Table S4.** Model evolution of pollen and ovule numbers based on the constrained tree. All the models tested for their evolution are shown, including Brownian Motion (BM), Early Burst (EB), and Ornstein-Uhlenbeck (OU). Akaike information criterion (AIC), corrected AIC (AICc), AIC weight (AICw), estimated evolutionary variance (σ²), and ancestral state (z_0) are shown for all models. The strength of selection (α) is estimated for the OU model. The best-fitting models, identified by the lowest AICc values, are shown in bold.

| Model | log-likelihood | AIC | AICc | σ² | α (OU) | z_0 | AICw |
| --- | --- | --- | --- | --- | --- | --- | --- |
| BM_pollen | -276.95 | 557.90 | 557.98 | 0.27 | - | 9.21 | 0.00 |
| EB_pollen | -276.95 | 559.90 | 560.06 | 0.27 | - | 9.21 | 0.00 |
| **OU_pollen** | **-266.05** | **538.12** | **538.27** | **0.46** | **0.10** | **9.30** | **1.00** |
| BM_ovule | -202.57 | 409.13 | 409.13 | 0.11 | - | 2.37 | 0.19 |
| EB_ovule | -202.57 | 411.13 | 411.13 | 0.11 | - | 2.37 | 0.07 |
| **OU_ovule** | **-200.19** | **406.39** | **406.54** | **0.14** | **0.04** | **2.45** | **0.74** |

**Table S5.** Results of GAM models testing the effects of biotic and abiotic variables on pollen and ovule production Test statistics (χ^2^), associated probabilities (p), and estimated degrees of freedom (df) are shown for each variable. Note that smooth terms with low predictive performance were penalised toward zero during model fitting. Significant effects are shown with bold font.

|  | Number of ovules | | |  | Number of pollen grains | | |
| --- | --- | --- | --- | --- | --- | --- | --- |
| Variable | (e)df | χ^2^ | p |  | (e)df | χ^2^ | p |
| Lifespan | 1.00 | 0.71 | 0.3980 |  | 1.00 | 0.02 | 0.8860 |
| Altitude | <0.01 | <0.01 | 1.0000 |  | **1.06** | **22.32** | **<0.0001** |
| Temperature seasonality | <0.01 | <0.01 | 1.0000 |  | **0.93** | **8.44** | **0.0017** |
| Precipitation seasonality | **0.84** | **4.57** | **0.0171** |  | <0.01 | <0.01 | 1.0000 |
| Climatic moisture index | **0.83** | **4.54** | **0.0184** |  | <0.01 | <0.01 | 1.0000 |
| Number of pollinators | <0.01 | <0.01 | 1.0000 |  | <0.01 | <0.01 | 1.0000 |
| Nutrient availability | <0.01 | <0.01 | 1.0000 |  | 0.46 | 0.85 | 0.1662 |
| Rooting conditions | 0.71 | 2.30 | 0.0684 |  | **1.73** | **11.72** | **0.0009** |

**Table S6.** ANOVA results and effect sizes for pollen and ovule numbers. For each factor, we estimated the sum of squares (Sum_Sq), degrees of freedom (Df), F-statistics (F_value), p values, effect size (Eta_squared), and 95 % confidence interval (CI_95). Significant results are highlighted in bold.

| Trait | Factor | Sum_Sq | Df | F_value | p_value | Eta_squared | CI_95 |
| --- | --- | --- | --- | --- | --- | --- | --- |
| Pollen | ploidy | 4989192300.58466 | 1 | 2.7314 | 0.1029 | 0.0376 | 0.95 |
| Pollen | mating | 44031251187.4489 | 1 | 24.1055 | **0.000005747*** | 0.2562 | 0.95 |
| Pollen | ploidy:mating | 1660640032.03896 | 1 | 0.9091 | 0.3436 | 0.0128 | 0.95 |
| Pollen | Residuals | 127862509390.471 | 70 | - | - | - | - |
| Ovule | ploidy | 150.023470862579 | 1 | 0.1728 | 0.6789 | 0.0025 | 0.95 |
| Ovule | mating | 0.205228211067151 | 1 | 0.0002 | 0.9878 | 0 | 0.95 |
| Ovule | ploidy:mating | 669.039256451957 | 1 | 0.7707 | 0.383 | 0.0109 | 0.95 |
| Ovule | Residuals | 60765.0852201855 | 70 | - | - | - | - |

**Table S7.** Summary of PGLS (OU) and OLS results with AIC comparisons based on the main tree. The table includes AIC values, intercept estimates, coefficients for ploidy (Ploidy_Coeff), and mating system (Mating_Coeff), their associated p-values (Ploidy_p, Mating_p), and interaction effects (Interaction_Coeff, Interaction_p). Significant p-values are shown with strike and bold font.

| Model | Trait | AIC | Intercept | Ploidy_Coeff | Ploidy_p | Mating_Coeff | Mating_p | Interaction_Coeff | Interaction_p |
| --- | --- | --- | --- | --- | --- | --- | --- | --- | --- |
| PGLS | Pollen | 1789.12 | 4649.69 | 7349.18 | 0.56 | 43006.68 | **0.00*** | -14636.33 | 0.41 |
| PGLS | Ovule | 705.19 | 20.69 | 5.27 | 0.52 | -3.47 | 0.71 | 1.04 | 0.93 |
| OLS | Pollen | 1793.99 | 12836.18 | -5057.89 | 0.76 | 63652.20 | **0.00*** | -20430.89 | 0.34 |
| OLS | Ovule | 716.60 | 33.38 | -10.80 | 0.35 | -8.08 | 0.49 | 12.97 | 0.38 |

**Table S8.** Summary of PGLS (OU) and OLS results with AIC comparisons based on the constrained tree. The table includes AIC values, intercept estimates, coefficients for ploidy (Ploidy_Coeff), and mating system (Mating_Coeff), their associated p-values (Ploidy_p, Mating_p), and interaction effects (Interaction_Coeff, Interaction_p). Significant p-values are shown with strike and bold font.

| Model | Trait | AIC | Intercept | Ploidy_Coeff | Ploidy_p | Mating_Coeff | Mating_p | Interaction_Coeff | Interaction_p |
| --- | --- | --- | --- | --- | --- | --- | --- | --- | --- |
| PGLS | Pollen | 1792.34 | 2276.87 | 80.96.54 | 0.55 | 50459.64 | 0.00* | -20038.79 | 0.3 |
| PGLS | Ovule | 707.28 | 22.17 | 2.82 | 0.74 | -3.72 | 0.71 | 2.95 | 0.81 |
| OLS | Pollen | 1793.99 | 12836.18 | -5057.89 | 0.76 | 63652.20 | 0.00* | -20430.89 | 0.34 |
| OLS | Ovule | 716.60 | 33.38 | -10.80 | 0.35 | -8.08 | 0.49 | 12.97 | 0.38 |
